# In pursuit of reconstructing missing human hands

**DOI:** 10.1093/nsr/nwad002

**Published:** 2023-01-09

**Authors:** Xuan Gong, Bai-Yang Sun, Qi-Ning Wang, Cai-Hua Xiong

**Affiliations:** Institute of Medical Equipment Science and Engineering, School of Mechanical Science and Engineering, Huazhong University of Science and Technology, China; Institute of Medical Equipment Science and Engineering, School of Mechanical Science and Engineering, Huazhong University of Science and Technology, China; College of Engineering, Peking University, China; Institute of Medical Equipment Science and Engineering, School of Mechanical Science and Engineering, Huazhong University of Science and Technology, China

## Abstract

Exploring bio-intelligence of human limbs could provide a new perspective for reconstructing missing limbs.

Human beings are the most intelligent living beings on Earth. Creating reconstructions of missing human limbs that can be naturally integrated into the human body is a lofty ambition and faces challenges in neuroscience, information science, mechanical and electronic engineering, and many other disciplines. Based on the development history of robotic prosthetic hands over the past 70 years, here we present a short review of the initial motivations of the main research branches in this research field and, even more importantly, the most recent state-of-the-art advancements and possible changes for future research directions. It shows that existing robotic prosthetic hands leave us far from the goal of using artificial hands to reconstruct missing motion abilities, which implies that comprehension of human hands is still to be enriched. Exploring and leveraging bio-intelligence of human hands could deepen the understanding of human hands, and provide a promising solution to achieve the goal.

Nowadays, hundreds of millions of people are suffering from limb mobility and function difficulties (for example, 24.12 million in China, 38.2 million in the USA; details in Supplementary Table 1), wherein a large portion is hand disability (Supplementary Table 1). From the common ancestor (the close representative species: *Ouranopithecus macedoniensis* [1]) of gorillas, chimpanzees and humans to Homo sapiens, it took millions of years for humans to evolve such dexterous hands, with 29 muscles driving 19 bones and 19 articulations [2]. The natural human hand is so dexterous that people have been studying it for decades and are still working towards a counterpart to reproduce its natural movements at will or to behave at will like it.

Since the 1950s scientists have attempted to replace and replicate the human hand with artificial counterparts [3] that were controlled by neural signals, like surface or invasive electromyography (EMG). For 70 years, even the most advanced prosthetic hands could only produce limited motion types (Fig. 1). State-of-the-art work (Fig. 1) mainly focuses on approaches drawing on artificial intelligence (AI) technologies, i.e. pattern-based control [4–7], via human-machine interfaces (HMIs). In these approaches, motion commands of the robotic prosthetic hands (RPHs) are outputs from the HMIs, and inputs of the HMIs are from neural signals of the residual hands. The patterns refer to both muscle activity patterns and hand kinematic patterns, and the HMIs associate them with each other. The HMIs themselves usually involve surface or invasive electrodes and an AI model that is to be trained on pre-collected data sets of neural signals and hand motions. These approaches succeeded in reproducing numbers of complex motions by exploiting the trained association between neural signal patterns and hand motion patterns, and made great advancements in addressing the dilemma between the rate of correctly identifying patterns [4] and the number of patterns [6]. However, partly due to the nature of complex AI models (e.g. large neural networks) and the limited comprehension of the inherent structure and characteristics of the musculoskeletal and nervous system of the human hand at present, such HMIs that highly rely on learning from data and purely statistical relationships are inevitably treated as a black box used to classify neural signal patterns and hand motion patterns.

**Figure 1.**
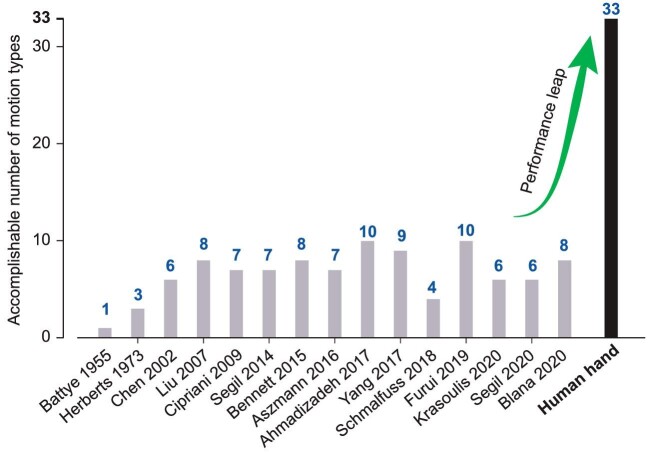
Achievable motion types of prosthetic hands over the past ∼70 years. Note that a series of similar works from the same research groups, as well as the studies that need further validation for amputees, might not all be listed. Detailed representatives and sources refer to Supplementary Table 2. The number of motion types of the human hand is classified according to a taxonomy of hand motions [10].

Before the black box was unboxed, it would be unlikely to leverage such classifiers to produce innumerable motion patterns like human hands do on the one hand, and to avoid the underlying bugs or unknown usage constraints for amputees using the RPHs that might crop up from time to time on the other. Uncertainties, either from the outside or the inside of the RPHs, could become severe problems rather than just regular bugs in software, as the RPHs must actually work with amputees in an intensively interactive and fully unstructured environment, which is still an open problem in research fields of robotics [8,9]. Could we separate the benefits of AI models and the flaws resulting from the black box? In fields where AI models are being widely applied, like computer vision and natural language processing, it turns out that large models could be well trained even before a complete mathematical explanation. However, the flaws might just be suppressed in certain situations and still risky in unknown ones, for example, being sensitive to adversary perturbations. Back to the scenario of RPHs, not only could the uncertainties be a problem but also the non-transparency for amputees. As long as RPHs cannot be used in the way human hands can, e.g. adhering to predefined motion patterns, an ideal counterpart of a natural hand will never be built.

Besides the principle-related challenges mentioned above, there are more practical and immediate issues relating to the black box. To classify movements, a rather complicated training process is necessary before actual implementation, which can sometimes be blind and tedious especially for deep-learning methods. When trained, the AI model can typically just reproduce the patterns it has learned, wherein the problem of generality comes. Intermediate motions between trained motion patterns, which demonstrate only subtle differences from the motion patterns and are however a distinct characteristic of natural human hands, would be a significant obstacle to making amputees feel like the RPHs were natural hands and accepting them into the proprioception system. Although the switching between patterns is an encouraging advancement that recently happened and a direct inspiration for producing motion patterns based on sequential combinations of trained patterns [6], the fashion of the switching is definitely becoming an issue of interest. It would be timely to capture the ever-changing motion patterns of natural hands, which means switching inside an AI model, e.g. a neural network could identify the pattern at present and predict some next pattern; this would be an advancement. Nevertheless, due to the temporal-spatial non-stationary nature of neural signals, it is challenging to develop RPHs that continuously switch among arbitrary motion patterns, which is definitely necessary to fulfilling the basics of human hand-motion abilities.

This whole assemblage of challenges could in part explain why even state-of-the-art RPHs, whether developed in academia or in industry and despite vast groups of very smart people working on them, are still far away from the goal of being adequate artificial hands that can recreate missing motion abilities: Fig. 1 illustrates numbers of motion types/patterns generated by RPHs in some representative work over the past 70 years, which means that even at the basic meaning of motion ability, i.e. motion type numbers, there is still a long way to go (Fig. 1) for the 30 motion patterns needed in daily activities according to a taxonomy of hand motions [10]. Advanced topics in human hand-motion abilities, e.g. dexterity for improvising tasks in daily activities, need to be studied and developed as well. All these factors could at least provide some insight into reasons why amputees are often very excited to try out new RPHs, but after a while are maybe not as thrilled about them, and will not use them in daily life. They might also provide insight into amputees’ opinions, such as thinking of RPHs as neurally manipulated joysticks that activate prescribed motions, feeling so tired of the long-term use of RPHs that they would rather use residual stumps [11,12], and imagining some sci-fi RPHs that could outperform human hands in some aspects, e.g. could crush apples.

Is there any other approach different from the mainstream HMI mentioned above that would possibly work or lay potential foundations for the goal of enabling amputees to control prosthetic hands at will just like using natural hands? After the Second World War, researchers attempted to directly control single-DOF (degree of freedom) prosthetic hands using human neural signals [3]. Robotic hands with five fingers mimicking the human hand in shape have been in development since about 40 years ago. Current devices are much lighter and can be used as hand prostheses due to the fact that they are controllable by neural signals. However, due to cognitive limitations, e.g. unknowns in a complex natural biomechanical system such as the human hand, and the lack of a design principle of the counterpart system based on cognitive research, the direct and proportional control approach using neural signals cannot be used to control multi-DOF prosthetic hands and produce motions dexterously. Some prior work, i.e. synergy-based or regression-based work, has suggested possible directions for this idea [13–15]. It has been shown that hand movements during manipulation can be approximated by lower dimensional ‘kinematic synergies’, which could be used to adaptively grasp objects with different shapes, with only one channel of surface electromyograph (sEMG) signal and a single DOF of hand closure/opening needed [14]. However, directly controlling RPHs with multiple DOFs like natural hands means, at least, producing motions much more complex than the aforementioned RPHs with AI models. In fact, although there exists some work that aims at developing AI models to continuously convert neural signals into motor commands, which has shown encouraging advancements in producing simultaneous motions of wrist rotation and hand closure/opening [15] (especially sufficient for continuous motion control of the arm and wrist [12]), these approaches are typically only applicable in limited DOFs for multi-finger motions; in contrast, the aforementioned approaches of classifying neural signals and hand motions as discrete patterns are shown to be more accurate, efficient and stable in reproducing multi-finger hand motions, as far as we know [11,12,15].

A shared puzzle underlying these issues is now clear: have we really understood how human hands are endowed with such superior motion abilities compared to those of developing RPHs? Can the methods of developing state-of-the-art artificial hands reversely help understand them? With such questions unanswered, comprehension of human limbs needs to be enriched. Humans ourselves of course need the neural musculoskeletal system to generate motions, which seems well known and less valuable in terms of further research. But it is only well known because we ourselves are human and we just use this mechanism to generate motions, we actually do not know how either, and that is where scientific research should be carried out. In fact, there are some physiological studies, some of which were carried out from an evolutionary perspective or a comparative one, that gather evidence and support such impressions with data and analysis, which should be considered in reconstructing missing human limbs and might provide some insight as to why human hands are more dexterous than the hands of other primates, whether there are some muscles unique or originating/functioning differently, how skeletal structure guarantees finger synergistic motions, and why neural activities drive corresponding hand motions. Human limbs, such as hands and feet, are biomechanical marvels representing a triumph of the most complex natural engineering in the body. Since the evolution of human bipedalism began ∼4 million years ago, human forelimbs were no longer needed for locomotion, and then became a highly intelligent biomechanical system with excellent grasping and manipulation abilities that were different from other primates [16]. The exceptional manipulation ability in humans is inseparable from the hand's complex musculoskeletal system, which is unique amongst primates [17–19]. The human hand looks like a bony puppet lashed together by ligaments. The neural mechanism underlying hand movements is also critical to the hand's dexterity. Unlike lower mammals, primates have evolved direct connections between neurons in cortical motor areas and spinal motoneurons, giving the cerebral cortex monosynaptic control over the motoneurons of the hand muscles and placing the hands ‘closer’ to the brain [2]. The recent evolution of the human hand is thus a direct result of the development of the central nervous system [19].

The musculoskeletal system of the human hand is very complex. There are inherent direct connections between human motor neurons and muscles driving hand motions. The musculoskeletal system carries some motion intelligence of the human hand. If the inherent characteristics are ignored, the progress of developing an artificial hand would be like a blind person trying to recognize an elephant just by touching it: the real whole scene can never be seen, and how the systems work can never be understood. The monkey's hands are similar to human hands in appearance, however far less dexterous than human hands, e.g. a monkey’s ability to use tools is far inferior to that of humans, due to the fact that a monkey’s musculoskeletal system is different [16,19]. If the motion intelligence of the human hand is not well understood, how can anyone ensure that the developed ‘hand’ is a counterpart of the human hand rather than the monkey hand?

Existing physiological studies of human hands are not enough, or well suited, for research on RPHs. Retrospecting human-hand evolution from not only the perspective of physiology but also of robotics and information science might be a direction for the field. Different levels of human hand-motion abilities need to be reconstructed, some of which have been shown to be empirically feasible with technologies of state-of-the-art RPHs: underactuated mechanical design for anthropomorphism, pattern recognition in pattern-based control for complex motion patterns, electrode implantation and functional electrical stimuli for feedback restoration and much more. In fact, like the concept of AI, bio-intelligence is an aim-oriented set of scientific foundations, technological tools and implementations. The aim is the replication of the superior abilities of human hands. AI promises to replicate the comprehensive cognitive abilities of humans, which is widely accepted as intelligence. However, in the field of RPHs, the aim is motion abilities rather than cognitive intelligence. Interdisciplinary research in this direction would definitely deepen our understanding of the bio-intelligence of human hands, which is defined by existing fossils, the structure of the musculoskeletal and nervous systems, and also by ongoing cutting-edge studies.

## Supplementary Material

nwad002_Supplemental_Files
